# A Patient-Centered Methodology That Improves the Accuracy of Prognostic Predictions in Cancer

**DOI:** 10.1371/journal.pone.0056435

**Published:** 2013-02-27

**Authors:** Mohammed Kashani-Sabet, Richard W. Sagebiel, Heikki Joensuu, James R. Miller

**Affiliations:** 1 Center for Melanoma Research and Treatment, California Pacific Medical Center and Research Institute, San Francisco, California, United States of America; 2 Molecular Cancer Biology Program, University of Helsinki, Helsinki, Finland; University of Connecticut Health Center, United States of America

## Abstract

Individualized approaches to prognosis are crucial to effective management of cancer patients. We developed a methodology to assign individualized 5-year disease-specific death probabilities to 1,222 patients with melanoma and to 1,225 patients with breast cancer. For each cancer, three risk subgroups were identified by stratifying patients according to initial stage, and prediction probabilities were generated based on the factors most closely related to 5-year disease-specific death. Separate subgroup probabilities were merged to form a single composite index, and its predictive efficacy was assessed by several measures, including the area (AUC) under its receiver operating characteristic (ROC) curve. The patient-centered methodology achieved an AUC of 0.867 in the prediction of 5-year disease-specific death, compared with 0.787 using the AJCC staging classification alone. When applied to breast cancer patients, it achieved an AUC of 0.907, compared with 0.802 using the AJCC staging classification alone. A prognostic algorithm produced from a randomly selected training subsample of 800 melanoma patients preserved 92.5% of its prognostic efficacy (as measured by AUC) when the same algorithm was applied to a validation subsample containing the remaining patients. Finally, the tailored prognostic approach enhanced the identification of high-risk candidates for adjuvant therapy in melanoma. These results describe a novel patient-centered prognostic methodology with improved predictive efficacy when compared with AJCC stage alone in two distinct malignancies drawn from two separate populations.

## Introduction

The art of prognosis has a long history, as physicians have attempted to understand the clinical behavior of disease. Ancient Egyptians estimated patient survival in order to arrive at an initial conclusion of either “a patient I will treat” or “a patient not to be treated” (the former with a chance to cure and the latter thought to be incurable). More recently, prognostic models have been developed using computerized analyses of large databases of patients with commonly recorded factors in order to predict outcome. In such factor-centered analyses, results are usually stated in terms of relative risks, odds ratios and P-values associated with each factor. In the realm of cancer, staging classifications are developed from these prognostic analyses and constitute the primary means of predicting patient outcomes and of making treatment decisions. However, they are not routinely the products of patient-centered analyses. Assigning a 5-year survival probability to a group of patients in a particular stage of a given malignancy is not the same as providing a separately tailored prognostic probability for each individual patient.

Patient-centered analyses take a different approach. Prognostic conclusions are stated in terms of an individual patient's probability of experiencing and/or the time required to experience some salient event, such as recurrence or death. Prognostic factors do help to determine these probabilities and elapsed times, but the factors, themselves, are not the primary focus of the analyses. Patient-centered success measures must reflect the accuracy of individual probabilistic predictions rather than the relative potency of the prognostic factors. In addition, patient-centered prognoses must identify and exploit the most relevant factors that can drive clinical decisions for an individual patient. The risk of progression or death may best be predicted by addressing factors beyond those incorporated into the staging classification and by analyzing available prognostic factors in specifically novel ways. In this manuscript, we developed a patient-centered prognostic methodology and applied it to established databases of melanoma and breast cancer patients to determine its predictive accuracy, when compared to predicting strictly on the basis of initial stage.

## Materials and Methods

### Ethics Statement

This prognostic analysis was approved by the institutional review boards at the University of California, San Francisco, and at the California Pacific Medical Center. The analysis was based on a chart review of the majority of patients entered into the datasets. Consequently, it was deemed minimal risk by these review committees, and informed consent was not required. Written informed consent was obtained from the patients whose tissues were tested as part of the analysis. These procedures were approved by the aforementioned institutional review boards.

### Study Populations

We accumulated a cohort of 1,222 United States patients, diagnosed with primary cutaneous melanoma between 1971 and 2006, whose demographic composition appears in Table S1. The mean and median follow-up times were 7.93 years and 7.44 years, respectively.

In addition, we had access to a previously described [1] dataset of 1,225 breast cancer patients from Turku, Finland, with a mean and median follow-up of 9.97 and 8.5 years, respectively. The demographic composition of the breast cancer cohort appears in Table S2.

### Analysis of Prognostic Factors

#### Melanoma

Fifteen prognostic factors were recorded at the time of diagnosis of primary cutaneous melanoma and distributed into two prognostic factor groups. The first factor group comprised six factors, including three histological factors incorporated into the current AJCC staging classification (i.e., tumor thickness, ulceration, and mitotic rate) [2], and three clinical factors included in analyses of the AJCC melanoma staging committee (i.e., age, gender, and tumor site) [3], [4]. The following nine histological factors were included in a second factor group: histological subtype, Clark level, presence or absence of microsatellites, vascular involvement, regression, degree of tumor vascularity, level of tumor infiltrating lymphocytes, number of positive lymph nodes, and the within-subgroup initial AJCC stage. The potential prognostic significance of these factors was previously reviewed [5]. The manner in which these additional prognostic factors were defined, measured, and coded was described previously [6], [7].

The prognostic impact of nine molecular factors (NCOA3, SPP1, RGS1, WNT2, FN1, ARPC2, PHIP, POU5, and p65 subunit of NF-κB), constituting a third factor group, was examined in tissues from 375 of the 1,222 melanoma patients using immunohistochemical analysis. The individual role of several of these markers in melanoma progression, including the methods used for immunohistochemical staining and scoring, was previously described [8]–[11]. The prognostic significance of several of these molecular factors has been validated in other tissue sets or by other investigators [10], [12]–[14].

#### Breast Cancer

We performed a similar analysis in our cohort of 1,225 breast cancer patients. The available prognostic factors were divided into the following three groups: the first factor group included patient age, anatomical location of the primary tumor within the breast, size of the primary tumor along its longest dimension (in millimeters), mitotic count, and ulceration of the primary tumor. The second factor group consisted of the following twelve factors: primary tumor type (ductal or lobular), tumor grade, necrosis, tubule formation, nuclear pleomorphism, inflammation, estrogen receptor level (fmol./mg.), progesterone receptor level (fmol./mg.), bilaterality, T scale value, N scale value, and M scale value. The third factor group consisted of the following two factors: radiation therapy (yes or no), and type of adjuvant therapy, if any.

### Statistical Analysis

To develop a patient-centered prognostic algorithm for disease-specific death within 5 years of diagnosis, both the 1,222 melanoma and 1,225 breast cancer patients were first stratified into three risk-defined subgroups, based on AJCC stage at diagnosis, if available, or T, N, and/or M stage. In the melanoma cohort, this resulted in a low-risk subgroup containing 503 patients, an intermediate-risk subgroup containing 423 patients, and a high-risk subgroup containing 296 patients. In the breast cancer cohort, the low-risk subgroup encompassed 552 patients, the intermediate subgroup comprised 387 patients, and the high-risk subgroup included 286 patients. Stratifying both samples into these three subgroups served to maintain sufficient subgroup sizes to support stable statistical estimates, while preserving the rank order of 5-year survival rates by stage inherent in each cohort.

Then, each prognostic factor was transformed, separately within each risk subgroup, via the Scale Partitioning and Spacing Algorithm (SPSA) into a corresponding Univariate Impact Reflecting Index (UIRI), as described in Methods S1.

For each of the nine prognostic factor group and patient risk subgroup combinations, an individualized prognostic algorithm was developed (described in Methods S1). The algorithm was based on the logistic regression analysis whose dependent variable was experience or non-experience of disease-specific death within five years of diagnosis and whose independent variables were the UIRI values calculated for the risk factors and patient subgroup constituting that combination. A composite prognostic algorithm was then constructed by merging the logistic regression outputs of the three patient risk subgroups, when all risk factors (i.e., their UIRI values) were used as independent variables of the regression.

The prognostic efficacy of the composite algorithm was assessed using three measures: the AUC generated by a receiver operating characteristic (ROC) analysis; its mean individual probabilistic prediction error; and its minimally achievable misclassification rate (the latter two are defined in Methods S1). All reported P values are two-sided.

## Results

To develop a patient-centered approach, we analyzed a cohort of 1,222 patients with primary cutaneous melanoma (Table S1) and a separate cohort of 1,225 patients with breast cancer (Table S2).

### A Tailored Prognostic Model for Melanoma

**Figure 1:**
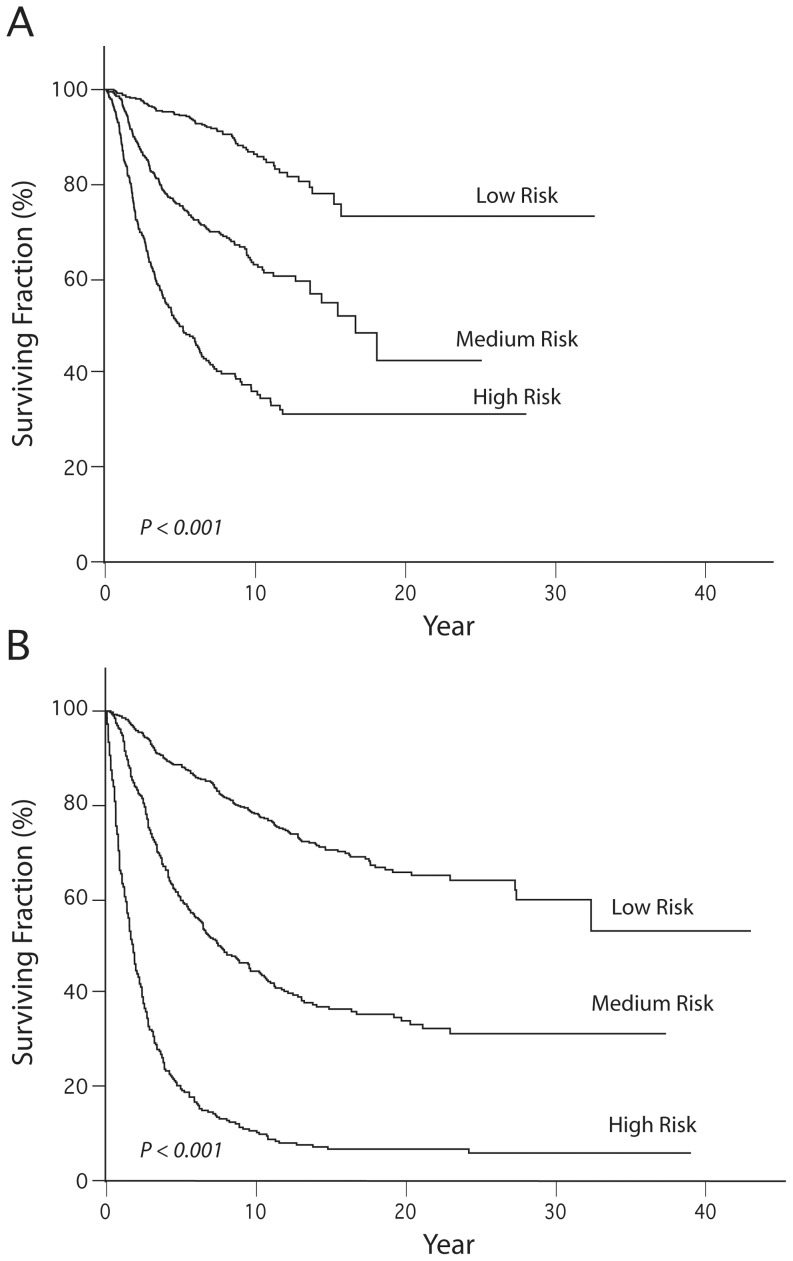
Panel A. Kaplan-Meier analysis of DSS by prognostic subgroup in the melanoma cohort. Panel B. Kaplan-Meier analysis of DSS by prognostic subgroup in the breast cancer cohort.

Initially, we stratified our melanoma cohort, based primarily on initial stage, into three patient subgroups. The low-risk subgroup had a 94.6% 5-year disease-specific survival (DSS), the intermediate-risk subgroup had a 75.4% 5-year DSS, and the high-risk subgroup had a 49.3% 5-year DSS. The three subgroups had significantly different survival characteristics, when assessed by 5-yr DSS (Kruskal-Wallis test corrected for tied observations, P<0.001) and by Kaplan-Meier analysis (Log-rank test, P<0.001, Fig. 1A).

**Figure 2:**
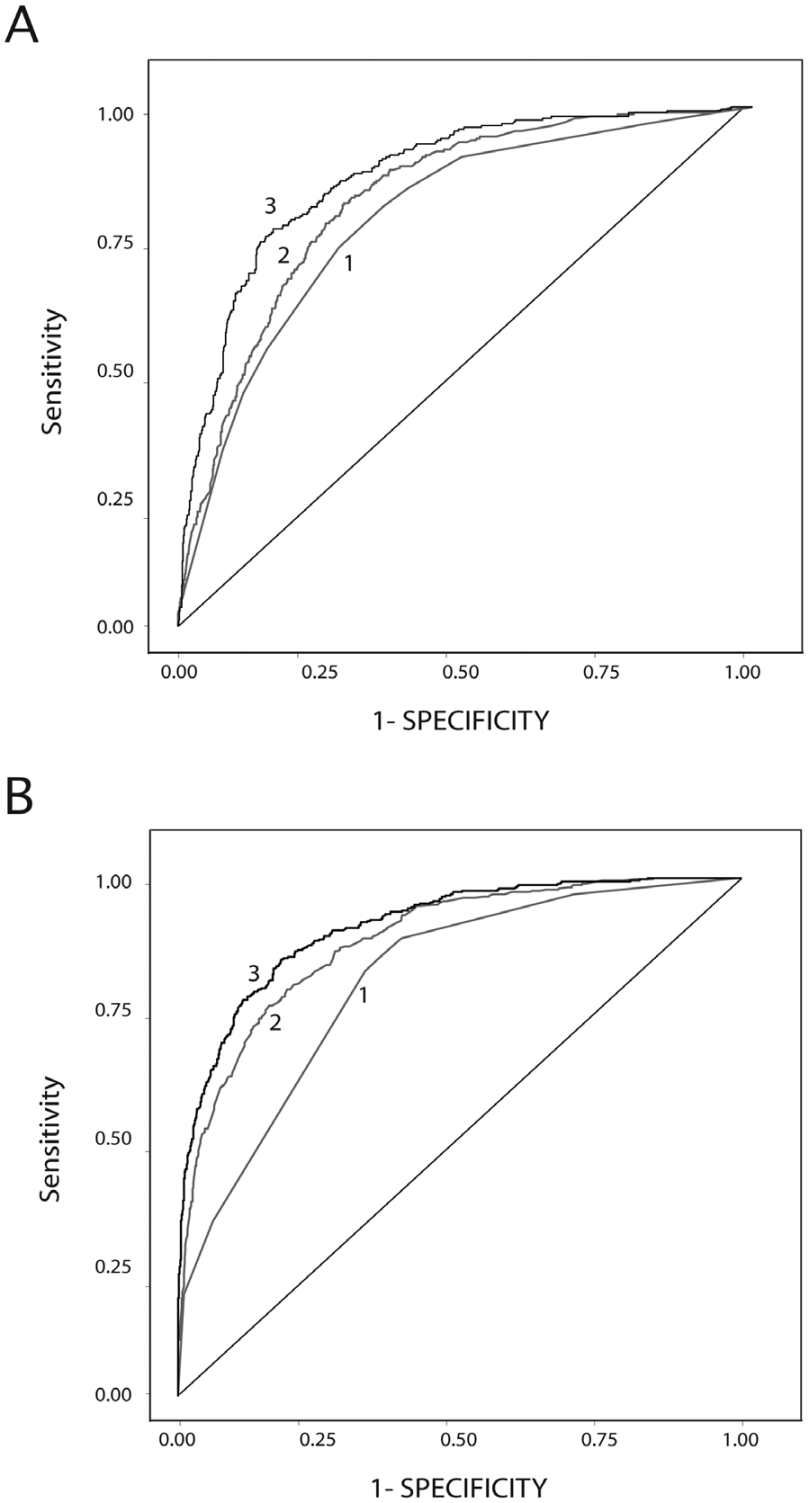
Panel A. ROC plots of 5-year melanoma-specific death probabilities estimated by different logistic regression analyses. Panel B. ROC plots of 5-year breast cancer-specific death probabilities estimated by different logistic regression analyses. In each panel, curve 1 represents the ROC plot using initial AJCC stage (unstratified), curve 2 the ROC plot stratified by AJCC stage, and curve 3 the ROC plot determined by the composite weighted index.

For each prognostic factor group and patient subgroup we developed a separate prognostic algorithm that best predicted 5-year disease-specific death. Separate algorithms were merged into a single, composite algorithm for each risk subgroup. Each composite algorithm produced a corresponding composite prognostic index. Values of this index were individual probabilities of 5-year disease-specific death assigned by the composite prognostic algorithm to each patient. Under an ROC analysis, the composite index generated an AUC of 0.867 (Fig. 2A). It was able to correctly predict 84.0% of the 5-year disease-specific events, resulting in a misclassification rate of 16.0%.

We compared the prognostic efficacy of the composite index with several other prognostic methodologies. Initially, we assessed the six routinely available prognostic factors by estimating individual probabilities of 5-year disease-specific death from a multiple logistic regression of these factors. This produced an AUC of 0.762, and a misclassification rate of 21.2% (Table 1).

**Table 1:** Comparison of predictive accuracy achieved in melanoma through differing prognostic methodologies (N = 1,222).

Prognostic Methodology	AUC	Mean Reduction	T value	P value
Six traditional prognostic factors (unstratified logistic regression)	0.762	N/A	N/A	N/A
AJCC stage (dummy variable logistic regression)	0.787	0.015	3.67	<0.001
Six traditional prognostic factors (logistic regression stratified by AJCC stage)	0.823	0.016	4.35	<0.001
Composite index (logistic regression, stratified by AJCC stage, incorporating 18 additional factors)	0.867	0.033	9.62	<0.001

Note: T values and accompanying 2-tail P values refer to reductions in mean absolute probabilistic error achieved relative to the prognostic methodology tabled in the line immediately above, where each matched-pair T test is applied to the indicated 1,222 matched pairs of individual probabilistic prediction errors.

Next, we performed a dummy-variable logistic regression using AJCC stage, alone, to assign 5-year disease-specific death probabilities in our melanoma sample and determined its prognostic efficacy. This analysis yielded an AUC of 0.787 (Fig. 2A and Table 1) and reduced mean absolute probabilistic prediction error (matched-pairs T-test, P<0.001, Table 1).

Then, we included the six prognostic factors and used initial AJCC stage to stratify the 1,222 patients into the three risk subgroups. The individual probability estimates generated by the multiple logistic regression analyses for each subgroup were merged, resulting in an AUC of 0.823, and further reduced mean absolute probabilistic error (matched-pairs T-test, P<0.001, Table 1).

We then incorporated the eighteen additional prognostic factors and formed the composite algorithm described above to generate the final prognostic index. Enhancing the model in these ways increased the AUC to 0.867 and further reduced the mean absolute probabilistic error (matched-pairs T-test, P<0.001, Fig. 2A and Table 1).

We then constructed a separate weighted index designed to reflect the relative predictive potency of each prognostic factor in each risk subgroup (Table S3). Thus, tumor thickness, mitotic rate, tumor vascularity, RGS1 expression level, and FN1 expression level were uniformly potent predictors, with positive weights in all of the three subgroups.

### A Tailored Prognostic Model for Breast Cancer

We used the identical procedure to develop personalized predictions of 5-year DSS for breast cancer patients, using data from our cohort of 1,225 patients. We stratified the overall cohort into three risk subgroups, based on the AJCC staging criteria for breast cancer. The low-risk subgroup had a 88.6% 5-year DSS, the intermediate-risk subgroup had a 60.2% 5-year DSS, and the high-risk subgroup had a 19.9% 5-year DSS. The three prognostic subgroups had significantly different survival characteristics, when assessed by 5-yr DSS (Kruskal-Wallis test corrected for tied observations, P<0.001) and by Kaplan-Meier analysis (Log-rank test, P<0.001, Fig. 1B).

Application of the patient-centered approach to breast cancer patients generated an AUC of 0.907 (Fig. 2B). The final composite prognostic index developed for breast cancer was able to correctly predict 84.1% of the 5-year disease-specific deaths, resulting in a misclassification rate of 15.9%.

The initial factor-centered analysis consisted of five prognostic factors that were as comparable as possible to the factors used in the melanoma analysis (except for gender, as all patients were women). Combining these factors via logistic regression and developing an individually tailored probability of 5-year disease-specific death resulted in an AUC of 0.743 (Table 2).

**Table 2:** Comparison of predictive accuracy achieved in breast cancer through differing prognostic methodologies (N = 1,225).

Prognostic Methodology	AUC	Mean Reduction	T value	P value
Five prognostic factors (unstratified logistic regression)	0.743	N/A	N/A	N/A
AJCC stage (dummy variable logistic regression)	0.802	0.052	7.08	<0.001
Five prognostic factors (logistic regression stratified by AJCC stage)	0.880	0.064	11.69	<0.001
Composite index (logistic regression, stratified by AJCC stage, incorporating 14 additional factors)	0.907	0.037	9.77	<0.001

Note: T values and accompanying 2-tail P values refer to reductions in mean absolute probabilistic error achieved relative to the prognostic methodology tabled in the line immediately above, where each matched-pair T test is applied to the indicated 1,225 matched pairs of individual probabilistic prediction errors.

Next, we performed a dummy-variable logistic regression using AJCC stage, alone, to assign 5-year disease-specific death probabilities due to breast cancer and determined its prognostic efficacy. This analysis yielded an AUC of 0.802 (Fig. 2B) and reduced mean absolute probabilistic prediction error (matched-pairs T-test, P<0.001, Table 2).

We then stratified the cohort using the three prognostic subgroups with distinct DSS. The individual probability estimates generated by the multiple logistic regression analyses for each subgroup were merged, resulting in an AUC of 0.880 and a further reduced mean absolute probabilistic error (matched-pairs T-test, P<0.001, Table 2).

Finally, we incorporated fourteen additional prognostic factors and formed the composite algorithm previously described to generate the final prognostic index. This procedure increased the AUC to 0.907 and further reduced the mean absolute probabilistic error (matched-pairs T-test, P<0.001, Fig. 2B and Table 2).

A separate weighted index similarly identified prognostic factors that were relatively potent predictors of 5-year disease-specific death in each risk subgroup (Table S4). Thus, mitotic rate and tumor grade were uniformly potent predictors, with positive weights in all of the three subgroups.

### A Split-Sample Validation of the Tailored Prognostic Methodology in Melanoma

In order to ascertain the reliability of the procedure used to construct our composite prognostic algorithm, we randomly split our sample of melanoma patients into a training subsample of 800 and a validation subsample of the remaining patients. Patients in the two subsamples were divided into three separate risk subgroups, using exactly the same criteria used to stratify patients in the total sample.

Next, we constructed a composite algorithm from the training subsample, using the same procedure applied to the entire cohort. This algorithm was quite similar to the algorithm produced for the total sample. The composite index generated by the composite prognostic algorithm constructed from the training subsample was found to be superior to the corresponding probabilistic indices derived from the six routinely available prognostic factors and from initial AJCC stage in both the training and validation subsamples by ROC analysis (data not shown).

Finally, we compared the prognostic efficacies achieved by the composite algorithm, when applied to the training and validation subsamples. When applied to the 800 patients in the training subsample, it achieved an AUC of 0.853. When applied to the remaining patients in the validation subsample, the same composite algorithm achieved an AUC of 0.789. Thus, the algorithm developed from the training subsample preserved 92.5% of its prognostic efficacy, as measured by AUC, when applied to the validation subsample.

### Utility of Tailored Prognostic Methodology for Identifying Patients Subsets for Adjuvant Therapy

**Figure 3:**
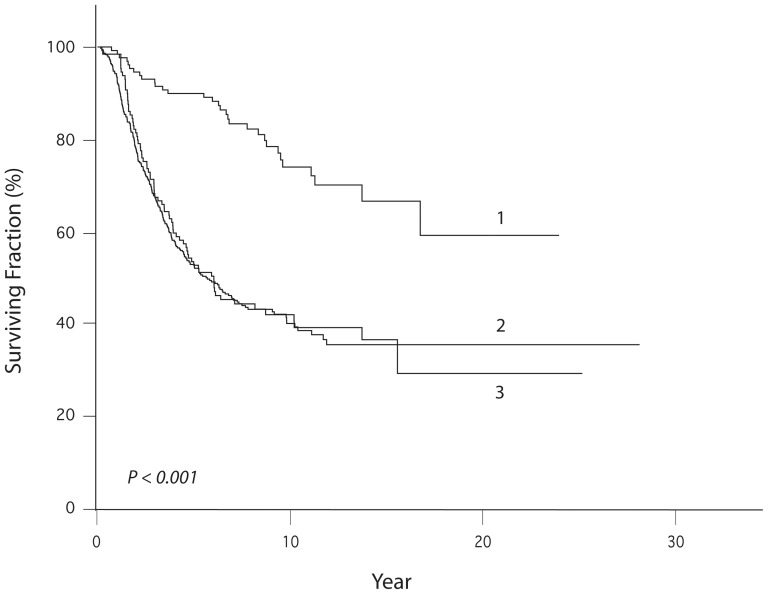
Kaplan-Meier analysis of DSS of high-risk patients identified by traditional eligibility for high-dose IFN only (curve 1), those identified by both criteria (curve 2), and those identified by the tailored prognostic model only (curve 3).

We then aimed to assess whether the tailored methodology could be utilized to identify specific prognostic patient subsets for systemic adjuvant therapy. High-dose interferon alpha (IFN) has been the standard adjuvant therapy for melanoma for over a decade. The traditional eligibility criteria for IFN [15]–[17] include patients with thick primary melanoma (greater than 4.0 mm thick) or node-positive disease. Using these criteria, we identified 492 patients in our melanoma cohort eligible for IFN treatment. We then identified an identical number of patients using our methodology with the highest individual probabilities of 5-year disease-specific death (excluding stage IV patients). These two subsamples were combined, and subsequently partitioned into three mutually exclusive subsets: 129 patients identified only by standard IFN eligibility criteria (group 1); 363 patients identified by both criteria (group 2); and 129 patients identified only by our methodology (group 3). Their survival was analyzed using Kaplan-Meier analysis. Whereas the DSS of groups 2 and 3 was not significantly different, the DSS of group 1 was significantly longer compared with either group 2 or 3 by (Fig. 3, log-rank test, P<0.001).

## Discussion

In this manuscript, we describe a patient-centered methodology to determine the prognosis associated with two common and potentially fatal cancers. We demonstrate that use of this approach results in significant improvements over the use of standard prognostic methodologies, when predictive efficacy is measured using AUC, probabilistic prediction errors, and misclassification rates in the prediction of 5-year death due to melanoma or breast cancer.

Use of our tailored prognostic approach resulted in AUC increases in predicting both 5-year cancer-specific deaths. We also demonstrate that use of this methodology results in the improved identification of high-risk candidates for adjuvant therapy in melanoma.

We achieved these improvements: (i) by first stratifying patients into separate risk groups according to initial stage and by then executing analyses, separately, for each group; (ii) by pre-converting all prognostic factors into comparably calibrated indices (UIRIs); (iii) by handling missing observations in a manner that does not require eliminating patients with sparse data from the analysis; and (iv) by incorporating additional prognostic factors not routinely captured in staging schemes, using these same three methodological devices.

In addition, our patient-centered approach is different from traditional prognostic analyses in a number of other ways. Traditional analyses typically focus on the relative prognostic potency of various factors using multivariate Cox or logistic regression. Yet possessing independent statistical significance does not guarantee that a factor will be prognostically useful for an individual patient [18]. In addition, staging schemes typically provide a survival estimate over a defined time period (e.g., 5- or 10-year survival) for all patients in a distinct substage of the cancer. By contrast, our approach converts prognostic output into tailored individual probabilities of some salient event, such as 5-year disease-specific death. This is the essence of the patient-centered approach. It focuses on individual patient outcomes rather than on the comparative potency of specific prognostic factors. Furthermore, it generates a separate probability of 5-year disease-specific death for each individual patient. It represents a shift in focus from the specific prognostic factors present in certain subgroups of patients to individual patient outcomes. While the role played by prognostic factors remains crucial, the factors now serve as the basis on which individually tailored patient probabilities are calculated. Prognostic factors are no longer the focus of the analysis in terms of which final conclusions are stated.

Since prognostic research usually focuses on identifying factors that provide statistically independent impact with a significant P value, whether or not alternative analytical procedures can improve prognostic efficacy at the level of individual patient outcomes is infrequently discussed and rarely demonstrated. Here we demonstrate the improvement in AUC achieved by our patient-centered prognostic approach, when compared with the use of AJCC stage in two different malignancies.

Developing tailored prognostic models is an important goal that has been examined by other groups. Cochran et al. [19] identified factors that emerged from logistic regression in a dataset of 1,042 melanoma patients, and developed individualized probabilistic risk estimates. Recently, the AJCC Melanoma Task Force developed an electronic tool to predict survival of localized melanoma using multivariate Cox regression analyses of five routinely available prognostic factors [20]. The survival estimates developed in a dataset of 14,760 patients were validated in an independent cohort of 10,974 patients. Significant procedural differences preclude comparisons with the patient-centered methodology described here. Importantly, no details were provided regarding the prognostic efficacy of their approach. However, in our cohort, the patient-centered approach was superior in prognostic accuracy when compared with the use of routinely available prognostic factors, alone.

Based on the results presented here, our patient-centered methodology may be of broad-based utility in making individually tailored prognoses for other cancers, as well as for other chronic diseases with significant morbidity. We utilized this methodology to improve prognostic accuracy and risk assessment for adjuvant therapy, but the same approach could also be used to identify patients with differential response to therapy. This may be especially relevant in the current debate to limit financial resources for health care. Methodologies that improve prognostic accuracy might also be useful in identifying patients who would benefit from receiving expensive and/or toxic therapies for chronic medical conditions.

Our prognostic approach enables the determination of individualized prognoses, even when values for many factors are missing. While it is helpful to have information for all prognostic factors, this is not practical for each individual patient. The patient-centered approach enables the determination of an individual's prognosis, based on whatever data are available. This is in contrast to a typical multivariate logistic or Cox regression, in which complete information on all prognostic factors is typically required for a given patient to be included in the analysis. In addition, our methodology identifies factors of greatest prognostic significance to distinct risk subgroups of patients and suggests which factors (that may be missing) would be most useful to include in a patient's pathology report (and prognostic assessment).

Datasets for the two malignancies selected to illustrate our patient-centered methodology were not population based. While population-based datasets are preferable in factor-centered analyses, it is more important in the patient-centered approach to identify patients who are prognostically “similar” to a particular patient whose prognosis is being determined. This distinction is another of the salient implications of moving from a strictly factor-centered to a patient-centered approach. However, in order to compile a comprehensive set of reference strata containing “similar” patients, it will be necessary to replicate this methodology in larger datasets that sample multiple strata of a general population with a given malignancy.

An important limitation of our patient-centered methodology is the possibility of statistical over-fitting. The same devices incorporated in the methodology that contribute to its improved prognostic accuracy also risk over-fitting the prognostic algorithm to whatever empirical observations are used as training data. To compensate for this, built-in protections against over-fitting include the admissibility criteria applied before introducing a candidate prognostic factor into the analysis and the minimum partition sizes established by the algorithm-generating procedure.

It is important to note that much of the improvement in predictive accuracy achieved by our methodology cannot reasonably be attributed to over-fitting. A substantial portion was realized simply by analyzing the modest number of routinely available prognostic and staging parameters in a different manner, prior to incorporating additional factors within the analyses (rows 1 and 2 vs. row 3 in Tables 1 and 2, respectively).

We have departed from the traditional approach to validating individual prognostic markers in which separate training and validation cohorts are used. Rather, we have developed a novel methodology, specifically designed to make prognostic predictions at the individual patient level. This methodology was then shown to improve prognostic accuracy (when compared with initial stage) in two data sets drawn from distinct populations and involving different cancers. In addition, a split-sample reliability analysis of the melanoma cohort revealed that a significant proportion (greater than 90%) of the prognostic accuracy achieved was retained in the validation subsample. Ultimately, however, our methodology would need to be applied to even larger data sets (several thousands of patients) both to mitigate excessive over-fitting and to produce a practically useful composite prognostic algorithm that could be used to make individual patient predictions.

Our study differs in its focus from important recent studies aimed at measuring the improvements in prognostic efficacy realizable from adding new biomarkers, especially when AUC is inadequate in its ability to detect changes in absolute risk [21]–[24]. In the realm of cancer, these techniques have been used to assess breast cancer risk [25]. In our analysis, both the use of ROC plots and probabilistic prediction methods proved adequate to demonstrate the improved efficacy of our tailored prognostic methodology. More importantly, our methodology goes beyond measuring predictive improvements. It offers procedures and devices by which such improvements can be realized.

In conclusion, we have developed a methodology to assign individualized probabilities to a specified focal event (e.g. five-year disease-specific death). This approach resulted in significant improvements in predictive accuracy in two different malignancies when compared with the use of routine prognostic methodologies, and can be used to tailor discussions regarding prognosis and therapy for an individual patient.

## Supporting Information

Methods S1 **Additional methods not included in the main text.**
(DOC)

Table S1 **Clinical and histologic characteristics of the melanoma sample (N = 1,222).**
(DOCX)

Table S2 **Clinical and histologic characteristics of the breast cancer sample (N = 1,225).**
(DOCX)

Table S3 **Relative weights in differentiating predictive potency of prognostic factors included in the melanoma sample (N = 1,222).**
(DOCX)

Table S4 **Relative weights in differentiating predictive potency of prognostic factors included in the breast cancer sample (N = 1,225).**
(DOCX)
