# Correction: A Patient-Centered Methodology That Improves the Accuracy of Prognostic Predictions in Cancer

**DOI:** 10.1371/annotation/2db613f0-06cc-47e7-bde2-7c61bc792bb9

**Published:** 2013-10-21

**Authors:** Mohammed Kashani-Sabet, Richard W. Sagebiel, Heikki Joensuu, James R. Miller

The Competing Interests Statement contains information that is no longer accurate. The Competing Interests Statement should read: "Mohammed Kashani-Sabet owns stock in Melanoma Diagnostics, Inc., and James R. Miller III has ownership interest in MDMS, LLC. A patent application was filed regarding the patient-centered methodology described here. On the basis of prior work, patent applications were filed regarding the diagnostic and prognostic impact of the some of the markers described here, and were licensed to Myriad Genetics. This does not alter the authors' adherence to all of the PLOS ONE policies on sharing data and materials, as detailed online in the guide for authors." 

