# Supplementary material for: A Patient-Centered Methodology That Improves the Accuracy of Prognostic Predictions in Cancer
Source: PLoS One. 2013 Feb 27;8(2):e56435. doi: 10.1371/journal.pone.0056435 (PMC3584071; doi:10.1371/journal.pone.0056435)
Supplement: Methods S1 — Additional methods not included in the main text. (DOC) [file pone.0056435.s001.doc]

**Supplemental Methods**

We collected two distinct and completely unrelated samples to demonstrate our patient-centered methodology: 1,222 male and female United States patients diagnosed with cutaneous melanoma at UCSF between July, 1971, and November, 2006, and followed up until October, 2010; and 1,225 female patients diagnosed with invasive breast cancer in Turku, Finland, between April, 1945, and December, 1984, and followed up until April, 1996. The mean follow-up time of our melanoma training sample was 7.93 years. The mean follow-up time of our breast cancer training sample was 9.97 years.

We selected 5-year disease-specific death (or its complementary event, 5-year DSS) as our focal end-point for the following reasons: (i) to compare patient outcomes within our melanoma sample with those surveyed by the AJCC, in which 5-year survival rates were measured and reported;^4,6^ and (ii) to make appropriate comparisons between our two training samples.

A Traditional Factor-Centered Analysis

We began with a traditional factor-centered analysis. It was a standard logistic regression of routine prognostic factors typically available for each cancer. There were six traditional factors for melanoma patients, including three histological factors incorporated into the current AJCC staging classification (i.e, tumor thickness, ulceration, and mitotic rate),^4^ and three clinical factors included in prior analyses of the AJCC melanoma staging committee (i.e., age at diagnosis, gender, and tumor site). ^5,6^

We chose five factors for breast cancer patients that were as comparable as possible to the six factors for melanoma. These included three comparable histological factors (tumor size measured in millimeters along its major dimension, ulceration, and mitotic count) and two comparable clinical factors (age at diagnosis and tumor site). Since all breast cancer patients were female, patient gender could not provide any discriminating predictive power and, therefore, was not included. In each traditional analysis, missing observations on any prognostic factor were uniformly replaced with the mean of the non-missing values of that factor in that training sample.

We performed multivariate logistic regression analyses of both sets of traditional factors and generated from each set of regression coefficients an estimated probability of disease-specific death within five years for each patient in each training sample. Estimated probabilities were calculated as: EXP [B0 + (B1)(X1) + (B2)(X2) + … + (BK)(XK)]/{1+ EXP[B0 + (B1)(X1) + (B2)(X2) + … + (BK)(XK)]},

where EXP means to exponentiate the immediately following bracketed expression relative to the natural logarithm base; B0, B1, B2, … , BK are the numeric values of the regression coefficients estimated by the logistic regression analysis; and X1, X2, …, XK are the numerically coded values of an individual patient’s K prognostic factors. K was six in the melanoma training sample and five in the breast cancer training sample. Based on these estimated probabilities, the efficacy of the traditional factors in predicting disease-specific death within five years was ascertained, using ROC analysis and an estimate of the AUC.

In addition, individually tailored probabilities enabled the calculation of a probabilistic prediction error, which was determined as follows: each patient in each training sample was assigned a value of either 0 or 1 on the basis of whether that patient had actually survived or experienced disease-specific death within 5 years, respectively; the actual 0/1 value was subtracted from each patient’s individually estimated probability of disease-specific death within five years; and the absolute value of the difference was defined as that patient’s probabilistic prediction error. The relative accuracy of individual patient probabilities generated by any two different probability estimation techniques was uniformly assessed via a matched-pairs analysis of their respective probabilistic prediction errors (e.g., via a matched-pairs T test or a binomial sign test).

Moreover, individually tailored probabilities enabled the calculation of a minimally achievable misclassification rate as follows: rank order the patients in each training sample by decreasing estimated probability of disease-specific death within 5 years; select a probability in this rank order; declare that all patients whose 5-year disease-specific death probabilities are at least this value will die of metastatic cancer within five years and that all patients whose five-year disease-specific death probabilities fall below this value will survive for more than five years; count the number of patients in that training sample about whom correct declarations have been made; repeat this procedure for all but the lowest probability in the rank order; and identify the probability with the lowest misclassification rate.

Establishing a Base Case for Practicing Physicians

Practicing physicians typically rely upon a patient’s initial staging classification as the primary basis for making prognoses. The AJCC has established staging criteria for both melanoma and breast cancer. Individually tailored probabilistic predictions of 5-year disease-specific death can be obtained from a logistic regression analysis of AJCC stage in each cancer.

Because a staging classification is designed to stratify patients into separate sub-populations according to the stage of development of their cancer at the time of initial diagnosis, 0/1 dummy

variables may be defined for successive stages. A logistic regression analysis of these dummy variables, again using 5-year disease-specific death as the dependent variable, and again converting regression coefficients into individual patient probabilities by the same procedure outlined above, generates an alternative set of probabilistic predictions. This base case analysis assigns the incidence in the training sample of 5-year disease-specific death for each AJCC stage to every patient initially diagnosed in that stage. Individually tailored base case probabilities are also subjected to a ROC/AUC analysis. The base case AUC can be compared with the AUC generated by the traditional factor-centered analysis. The two minimally achievable misclassification rates can also be compared. The two sets of individually tailored

probabilities can be compared via a matched-pairs test of their respective probabilistic prediction errors.

Constructing the Patient-Centered Methodology

The six traditional AJCC prognostic factors in melanoma and the comparable five factors in breast cancer were first converted to corresponding Univariate Impact-Reflecting Index (UIRI) values via the Scale Partitioning and Spacing Algorithm (SPSA), as follows:

1. Choose a prognostic factor whose directional relationship with the focal event has already been established, historically. The chosen prognostic factor must assign at least two distinct raw measurement values to patients in the training sample. Raw measurement values must be numerically coded.

2. Divide the training sample into two separate subsamples: the focal subsample, containing those patients who actually experienced the focal event (disease-specific death within five years of diagnosis); and the complementary subsample, containing those patients who experienced the complementary event (survival for more than five years). Each subsample must contain at least one patient.

3.Perform a Mann-Whitney test on the two subsamples in terms of the chosen factor’s raw measurement values. This provides an initial admissibility check on the factor’s proper directionality. If higher levels of the prognostic factor are associated, historically, with a higher risk of experiencing the focal event, the focal subsample should possess systematically higher raw measurement values. Minimal admissibility is achieved, so long as the factor does not point in the historically wrong direction. When achieved, historical admissibility is thereby verified for the current training sample. When verified, it is the factor’s scale of numerically coded raw measurement values that will then be partitioned into subscales and spaced by SPSA.

4.Assuming historical admissibility, if the raw measurement scale contains only two values, or if only two distinct values are assigned to patients in the training sample, there is only one possible way to partition the scale. Further verification of the chosen factor’s proper direction of impact is now sought.

5.Assuming that the higher raw measurement value indicates higher risk, define the factor’s impact sensitivity as the proportion of those patients in the focal subsample who received the higher-risk raw measurement value.

6. Assuming that the lower raw measurement value indicates lower risk, define the factor’s impact specificity as the proportion of those patients in the complementary subsample who received the lower-risk raw measurement value.

7. The chosen factor’s impact sensitivity ( a “true positive” indication) must exceed the proportion of patients in the complementary subsample who received the higher-risk raw measurement value (a “false positive” indication); its impact specificity (a “true negative” indication) must exceed the proportion of patients in the focal subsample who received the lower-risk raw measurement value (a “false negative” indication); and the proportion of those patients receiving the higher-risk raw measurement value who actually experienced the focal event must exceed the proportion of those patients receiving the lower-risk raw measurement value who actually experienced the focal event.

8. It is easy to show that each of the above three requirements for proper directionality logically implies the other two, in the case of otherwise admissible prognostic factors. If not further verified in this manner, the chosen factor is inadmissible. It either points in no direction or it points in the wrong direction.

9 When proper directionality is so verified, and when both the focal and complementary subsamples contain a large enough number of patients, no further steps need be executed. These two values define the optimally partitioned scale for the chosen prognostic factor. Each partition contains one of the two values. Proceed to spacing the two partitions by assigning UIRI values. Otherwise, subsequent steps are required to partition the factor’s at-least-three-valued raw measurement scale.

10. All possible cut-points within the training sample of rank-ordered raw measurement values must be checked to further verify admissibility. If the raw measurement data contain N ordered distinct values, there are N – 1 potential cut-points separating adjacent pairs of values in the rank order.

11. An admissible cut-point is one that both subdivides the training sample into two subsamples of patients – each containing at least a minimum partition size – and preserves the historically determined direction of impact in the three senses described above. Every cut-point defines a conceptual two-by-two cross-tabulated frequency table. The first row of such a table contains patients whose raw measurement value falls below the cut-point. The second row contains patients with values that fall at or above the cut-point. The first column of such a conceptual table contains patients in the complementary subsample. The second column contains patients in the focal subsample. The admissibility of any cut-point is determined by constructing its conceptual two-by-two cross-tabulated frequency table and applying these admissibility criteria.

12. Assuming there exists at least one admissible cut-point, the optimal cut-point is the one among them that maximizes the mean of the impact sensitivity and impact specificity that it determines. If the maximum is not unique, choose the cut-point from the maximizing set whose minimum-sized partition is largest.

13. In the absence of any admissible cut-points, there is no optimum cut-point, the factor’s raw measurement scale is not partitioned, and that chosen factor is declared inadmissible.

14. The raw measurement scale is actually partitioned into two subscales by the optimum cut-point, assuming one is successfully identified. There is a lower subscale and a higher subscale. The training sample is actually partitioned into lower and higher subsamples, accordingly.

15. The preceding steps are then repeated, first for each lower subscale and its associated lower subsample, and then for each higher subscale and its associated higher subsample.

16. Successive repetitions continue, as long as optimum cut-points, optimum sub-cut-points, optimum sub-sub-cutpoints, and so forth, continue to be identified.

17. At each step in the process where the repeated procedure generates either a lower or a higher subscale or both, and when either or both subscales are subsequently partitioned, additional directionality checks are also required across partitionings. Failure to pass such an additional check serves to invalidate the corresponding subsequent partitioning, but it does not invalidate any prior, successful partitionings.

18. A chosen prognostic factor may be eliminated as inadmissible either because it points in no direction or because it points in the historically wrong direction or because it fails to generate at least two raw measurement scale partitions, each of sufficiently large subsample size.

19. The maximum number of scale partitions that can be generated by this SPSA procedure is the number of distinct raw measurement values assigned to patients in a training sample. This occurs when each distinct raw value occupies its own separate partition (subscale). In practice, however, our experience to date suggests that only a few known factors for predicting cancer produce more than four separate impact-reflecting scale partitions. It is true that some factors possess more than four values in their raw measurement scales (e.g., those measuring tumor size in millimeters), but their effective discriminating power generally supports no more than four impact-reflecting partitions (subscales), as determined by actually executing SPSA.

20. Univariate Impact-Reflecting Index (UIRI) values are designed to embody the appropriate “spacing” among optimally partitioned subscales of a chosen factor’s raw measurement scale. UIRI values are coded as non-negative numbers. The numbers are calculated to reflect the relative magnitude of impact (possibly causal, possibly only correlational) linking a chosen prognostic factor’s ordered subscales to the focal event. In the context of a logistic regression analysis, where each patient’s individually tailored prediction is the estimated probability of experiencing the focal event, the UIRI value assigned to every patient is the incidence of the focal event in the training sample among all patients whose values of the chosen factor fall into the same partition (subscale) into which that patient’s raw factor value falls. UIRI incidences are uniformly expressed as relative frequencies.

21. There still remains the question of how to deal with missing observations on the chosen factor. We account for missing or unavailable observations in a uniform manner. All such observations are collected in a single, additional partition (subscale), assuming at least a minimum partition size. A reasonable estimate of the probability that a patient whose raw measurement on the chosen factor is missing will experience the focal event is the relative frequency of such patients in the training sample who actually did experience it. The UIRI value assigned to patients with missing observations is this relative frequency. Adding an additional, missing-observation partition (subscale) assumes that no systematically different reasons for being missing are known. It is analogous to assuming that no systematically different reasons are known for producing censored observations in Kaplan-Meier and Cox regression analysis.

22. If a missing-observation partition (subscale) would be too sparsely populated (i.e., of less than the minimum partition size), it is not added. Instead, missing observations are grouped with whichever partition (subscale) containing non-missing observations minimizes the resulting alteration of the originally calculated Mann-Whitney statistic. This convention is designed to minimize its biasing impact on the analysis.

23. Minimum partition size is a variable parameter of the SPSA algorithm. It is set to a positive integer, depending on the total size of the training sample. Minimum partition sizes in the hundreds provide reasonably stable UIRI values, assuming that the training sample may be viewed as randomly selected.

24. The SPSA algorithm just outlined may be executed either on a total training sample or, separately, on stratified subsamples. Whenever possible, selective stratification generally produces more accurate individually tailored prognostic predictions.

Revised AJCC staging parameters^4^ were used to stratify the melanoma training sample into three subgroups: a low-risk group, encompassing stage 1a and 1b disease; a medium-risk group, including stage 2a, 2b, 3a and 3b patients; and a high-risk group, encompassing patients with stage 2c, 3c, and 4 disease at initial diagnosis. When a patient’s AJCC stage could not be determined, tumor thickness was used. Unless there was evidence of nodal or distant metastasis at diagnosis, T1 patients were classified as low-risk; T2 patients were classified as medium-risk; and both T3 and T4 patients were classified as high-risk. This stratification strategy preserved the rank order of 5-year DSS survival rates by stage reported by the AJCC ^4-6^ and preserved sufficient sample sizes within each subgoup to enable separate applications of the SPSA algorithm and separate logistic regression analyses. It also achieved highly significant differences in 5-year disease-specific death rates between the three subgoups (P<0.0001 by Kruskal-Wallis analyses corrected for tied observations).

The 2010 AJCC staging classification was used to stratify the breast cancer training sample into three subgroups: a low-risk group, encompassing all stage 0, 1a, 1b, and 2a patients; a medium-risk group, including all stage 2b, most stage 3a, and some stage 3b patients; and a high-risk group, encompassing some stage 3a, most stage 3b, and all stage 3c and 4 patients at initial diagnosis. When a patient’s AJCC stage could not be determined, the T, N, or M stages were used to assign the patients into one of the three risk subgroups**.** The precise stratification rule for breast cancer patients was as follows: high-risk, if either (M1) or (M0 and N at least 2) or (M0 and N1 and T at least 3); medium-risk, if (M0 and N1) or (M0 and N0 and T at least 3) or (M0 and N undefined and T at least 3): otherwise, low-risk. This stratification strategy preserved sufficient sample sizes within each subgroup to enable separate applications of the SPSA algorithm and separate logistic regression analyses. It also achieved highly significant differences in 5-year disease-specific death rates between the three subgroups (P<0.0001 by Kruskal-Wallis analysis corrected for tied observations).

The SPSA algorithm converted each of the AJCC six traditional melanoma prognostic factors into corresponding UIRI values. SPSA also converted the five comparable breast cancer prognostic factors into corresponding UIRI values. The SPSA algorithm was applied, separately, to low-risk, medium-risk, and high-risk melanoma subsamples and, separately, to low-risk, medium-risk, and high-risk breast cancer subsamples.

Next, a multivariate logistic regression analysis of either six or five UIRI values was executed, separately, in each risk subgroup of each training sample. The common dependent variable of all six analyses was 5-year disease-specific death. The individually tailored probabilistic predictions generated by the three separate analyses of risk subsamples comprising each training sample were then merged to obtain an individually tailored probability for each patient in that training sample. The prognostic efficacy of this stratified approach was determined by calculating its AUC, its probabilistic prediction errors, and its minimally achievable misclassification rate and comparing these with comparable outputs of the preceding two prognostic methodologies (i.e., the traditional factor-centered and the base case methodologies). Subsequently, we incorporated additional prognostic information into the final patient-centered methodology applied to each cancer.

We defined two additional factor groups in melanoma: a nine-factor group incorporating additional histological parameters (i.e., histological tumor type, Clark level, presence or absence of microsatellites, vascular involvement, regression, degree of tumor vascularity, level of tumor infiltrating lymphocytes (TIL), number of positive lymph nodes, and the within-risk-subgroup initial AJCC stage); and one incorporating nine molecular factors (NCOA3, SPP1, RGS1, WNT2, FN1, ARPC2, PHIP, POU5, and the p65 subunit of NF-κB). All of the additional factors were converted by SPSA into corresponding UIRI values, as previously described.

We then performed a stratified logistic regression analysis, using AJCC stage to stratify patients into the three risk subgroups, and using as independent variables UIRI values corresponding to the AJCC six traditional prognostic factors and UIRI values corresponding to the eighteen additional factors in the two additional factor groups This generated a composite prognostic algorithm that assigned an individually tailored probability of experiencing five-year disease specific death to each of the 1,222 patients in the melanoma training sample.

We defined two additional factor groups in breast cancer: a twelve-factor group incorporating mostly additional histological parameters (i.e., primary tumor type, tumor grade, necrosis, tubule formation, nuclear pleomorphism, inflammation, estrogen receptor, progesterone receptor, bilaterality, T scale value, N scale value, and M scale value); and a two-factor group indicating therapeutic treatment (i.e., radiation therapy and type of adjuvant therapy, if any). All of the additional factors were converted by SPSA into corresponding UIRI values, as described.

We then performed a stratified logistic regression analysis, using AJCC stage to stratify patients into the three risk groups, and using as independent variables UIRI values corresponding to the five traditional prognostic factors and UIRI values corresponding to the fourteen additional factors in the two additional factor groups. This generated a composite prognostic algorithm that assigned an individually tailored probability of experiencing five-year disease-specific death to each of the 1,225 patients in the breast cancer training sample.

Estimating Relative Potency Weights

Given that some factors may be more potent than others as predictors of 5-year disease-specific death, relative potency weights were estimated as follows for each of the nine factor group/risk subgroup combinations in each training sample:

1. Begin with a table of UIRI values. Suppose that the table contains M rows, one for each of the M patients in the factor group/risk subgroup combination, and N columns, one to hold UIRI values for each admissible prognostic factor. SPSA has guaranteed that there are no missing-data cells in the table.

2. The task is to identify a set of N non-negative proportional weights adding to 1.0 that reflect the relative predictive potency of the N factors.

3. Tailored individual patient probability of experiencing disease-specific death within five years of diagnosis was chosen as the principal prediction of our patient-centered methodology. Therefore, weights should be selected to render the weighted average of the N component individual probabilities (embodied, respectively, in the N UIRI values) as close as possible to the actual 0/1 experience of the focal event.

4. Minimizing the sum of the squared deviations is a convenient way to accomplish this.

5. A quadratic minimization problem based on non-negative weights and a single linear constraint (the weights must add exactly to 1.0) has just been defined. The Kuhn-Tucker conditions provide a necessary and sufficient solution to such a problem. From these, a workable solution algorithm was devised and reduced to computer code.

6. Applying this algorithm to our training samples of 1,222 melanoma patients and 1,225 breast cancer patients produced the two weight tables.

Identification of High-risk Patients for Adjuvant Therapy in Melanoma

From the dataset of 1,222 melanoma patients, disease-specific survival of the 492 patients meeting the eligibility criteria for the randomized trials of high-dose IFN (> 4.0 mm thick or node-positive disease) in the melanoma training sample was compared with the DSS of the 492 top-ranked patients in terms of their tailored individual probability of experiencing disease-specific death within five years of initial diagnosis (excluding stage IV patients). These two partially overlapping subsamples were combined (621 patients, in all) and then partitioned into three mutually exclusive subsets: 129 patients deemed eligible by the standard IFN eligibility criteria only (but not by our patient-centered methodology); 129 patients deemed eligible by our patient-centered methodology only (but not by the standard criteria); and the intersection subset containing 363 patients identified using both criteria. The differences in DSS among these three subsets were assessed by Kaplan-Meier analysis, using the log-rank test.
