# Supplementary material for: A Patient-Centered Methodology That Improves the Accuracy of Prognostic Predictions in Cancer
Source: PLoS One. 2013 Feb 27;8(2):e56435. doi: 10.1371/journal.pone.0056435 (PMC3584071; doi:10.1371/journal.pone.0056435)
Supplement: Table S1 — Clinical and histologic characteristics of the melanoma sample (N = 1,222). (DOCX) [file pone.0056435.s002.docx]

Table S1. Clinical and histologic characteristics of the melanoma sample (N=1,222).

| Demographic Variable |  | Number (percent) |
| --- | --- | --- |
| Age | < 60 years | 818 (66.9) |
|  | > 60 years | 404 (33.1) |
|  | Mean: 51.5 years |  |
|  | Median: 51 years |  |
| Gender | Male | 743 (60.8) |
|  | Female | 479 (39.2) |
| Anatomical location | Head and neck | 268 (22.0) |
|  | Trunk | 487 (40.1) |
|  | Extremity | 461 (37.9) |
| Histologic Tumor Type | Superficial spreading | 464 (48.5) |
|  | Nodular | 251 (26.2) |
|  | Acral | 43 (4.5) |
|  | Desmoplastic | 48 (5.0) |
|  | Lentigo maligna melanoma | 32 (3.3) |
|  | Other or not otherwise classified | 119 (12.5) |
| Initial T Stage | T1 | 392 (32.4) |
|  | T2 | 290 (23.9) |
|  | T3 | 229 (18.9) |
|  | T4 | 301 (24.8) |
