# Supplementary material for: A Patient-Centered Methodology That Improves the Accuracy of Prognostic Predictions in Cancer
Source: PLoS One. 2013 Feb 27;8(2):e56435. doi: 10.1371/journal.pone.0056435 (PMC3584071; doi:10.1371/journal.pone.0056435)
Supplement: Table S2 — Clinical and histologic characteristics of the breast cancer sample (N = 1,225). (DOCX) [file pone.0056435.s003.docx]

Table S2. Clinical and histologic characteristics of the breast cancer sample (N=1,225).

| Demographic Variable |  | Number (percent) |
| --- | --- | --- |
| Age | < 60 years | 680 (55.5) |
|  | > 60 years | 545 (44.5) |
|  | Mean: 57.6 years |  |
|  | Median: 57 years |  |
| Anatomical location | Central | 158 (15.3) |
|  | Diffuse | 42 (4.1) |
|  | Lateral | 619 (59.9) |
|  | Medial | 214 (20.7) |
| Tumor Grade | Low-grade | 272 (22.2) |
|  | Medium-grade | 541 (44.2) |
|  | High-grade | 412 (33.6) |
| Initial T Stage | T0 | 5 (0.4) |
|  | T1 | 344 (28.5) |
|  | T2 | 560 (46.3) |
|  | T3 | 150 (12.4) |
|  | T4 | 150 (12.4) |
| Adjuvant Therapy | Ovarian ablation | 59 (4.8) |
|  | Tamoxifen | 31 (2.5) |
|  | Chemotherapy | 14 (1.1) |
|  | (cyclophosphamide, methotrexate, and 5-fluorouracil) |  |
|  | Other | 9 (0.7) |
|  | None | 807 (65.9) |
|  | Unknown | 305 (24.9) |
| Locoregional Radiation Therapy | Given | 919 (75.0) |
|  | Not Given | 304 (24.8) |
