# Supplementary material for: A Patient-Centered Methodology That Improves the Accuracy of Prognostic Predictions in Cancer
Source: PLoS One. 2013 Feb 27;8(2):e56435. doi: 10.1371/journal.pone.0056435 (PMC3584071; doi:10.1371/journal.pone.0056435)
Supplement: Table S3 — Relative weights in differentiating predictive potency of prognostic factors included in the melanoma sample (N = 1,222). (DOCX) [file pone.0056435.s004.docx]

Table S3. Relative weights in differentiating predictive potency of prognostic factors included in the melanoma sample (N = 1,222).

| Prognostic Factor | Low-risk group | Intermediate-risk group | High-risk group |
| --- | --- | --- | --- |
| Mitotic rate | 0.7293 | 0.4923 | 0.5931 |
| Thickness | 0.2032 | 0.0965 | 0.4069 |
| Age | N/A | 0.4112 | N/A |
| Gender | 0.0675 | N/A | N/A |
| Factor Group Total | 1.0 | 1.0 | 1.0 |
| Histologic subtype | 0.2361 | N/A | N/A |
| Clark level | 0.6355 | N/A | N/A |
| Tumor vascularity | 0.1284 | 0.0052 | 0.4227 |
| TIL level | N/A | N/A | N/A |
| Microsatellites | N/A | N/A | 0.0011 |
| Vascular involvement | N/A | 0.6558 | N/A |
| AJCC initial stage | N/A | 0.339 | N/A |
| Positive node count | N/A | N/A | 0.5762 |
| Factor Group Total | 1.0 | 1.0 | 1.0 |
| SPP1 expression | N/A | 0.1852 | N/A |
| RGS1 expression | 0.5478 | 0.7139 | 0.3052 |
| FN1 expression | 0.4051 | 0.1009 | 0.5148 |
| PHIP expression | 0.0471 | N/A | 0.074 |
| POU5 expression | N/A | N/A | 0.106 |
| Factor Group Total | 1.0 | 1.0 | 1.0 |

NOTE: N/A means that the prognostic factor did not possess sufficient relative predictive potency within its factor group/risk subgroup combination to achieve a positive least-squares weight. Relative predictive potency refers to a factor’s capacity to differentially predict five-year disease-specific survival, compared to the other prognostic factors in the same factor group/risk subgroup combination. Prognostic factors that failed to achieve a positive least-squares weight in any risk subgroup were excluded from the table. The weights add to 1.0 for each prognostic factor group/risk subgroup combination.
