# Supplementary material for: A Patient-Centered Methodology That Improves the Accuracy of Prognostic Predictions in Cancer
Source: PLoS One. 2013 Feb 27;8(2):e56435. doi: 10.1371/journal.pone.0056435 (PMC3584071; doi:10.1371/journal.pone.0056435)
Supplement: Table S4 — Relative weights in differentiating predictive potency of prognostic factors included in the breast cancer sample (N = 1,225). (DOCX) [file pone.0056435.s005.docx]

Table S4. Relative weights in differentiating predictive potency of prognostic factors included in the breast cancer sample (N = 1,225).

| Prognostic Factor | Low-risk group | Intermediate-risk group | High-risk group |
| --- | --- | --- | --- |
| Mitotic count | 0.6407 | 0.6859 | 0.6746 |
| Tumor Size | 0.031 | 0.3141 | N/A |
| Age | 0.3283 | N/A | N/A |
| Tumor Site | N/A | N/A | 0.3254 |
| Factor Group Total | 1.0 | 1.0 | 1.0 |
| Tumor grade | 0.497 | 0.6026 | 0.3391 |
| Necrosis | 0.4066 | 0.3974 | N/A |
| Pleomorphism | N/A | N/A | 0.437 |
| ER status | N/A | N/A | 0.056 |
| PR status | N/A | N/A | 0.1101 |
| T stage | 0.0467 | N/A | N/A |
| N stage | 0.0497 | N/A | N/A |
| M stage | N/A | N/A | 0.0578 |
| Factor Group Total | 1.0 | 1.0 | 1.0 |
| Radiation therapy | 0.8472 | 0.2595 | 0.5554 |
| Adjuvant therapy | 0.1528 | 0.7405 | 0.4446 |
| Factor Group Total | 1.0 | 1.0 | 1.0 |

NOTE: N/A means that the prognostic factor did not possess sufficient relative predictive potency within its factor group to achieve a positive least-squares weight. Relative predictive potency refers to a factor’s capacity to differentially predict five-year disease-specific survival, compared to the other prognostic factors in the same factor group and across factor groups. Prognostic factors that failed to achieve a positive least-squares weight in any risk subgroup were excluded from the table. The weights add to 1.0 for each prognostic risk subgroup.
